# Supplementary material for: Association between oral microbiome and seven types of cancers in East Asian population: a two-sample Mendelian randomization analysis
Source: Front Mol Biosci. 2023 Nov 21;10:1327893. doi: 10.3389/fmolb.2023.1327893 (PMC10702768; doi:10.3389/fmolb.2023.1327893)
Supplement: Supplementary file 1 [file DataSheet1.ZIP › Supplementary Materials/MR plots for tongue/tongue═╝/Lung cancer/pheno.1699_to_lung cancer_forest.pdf]

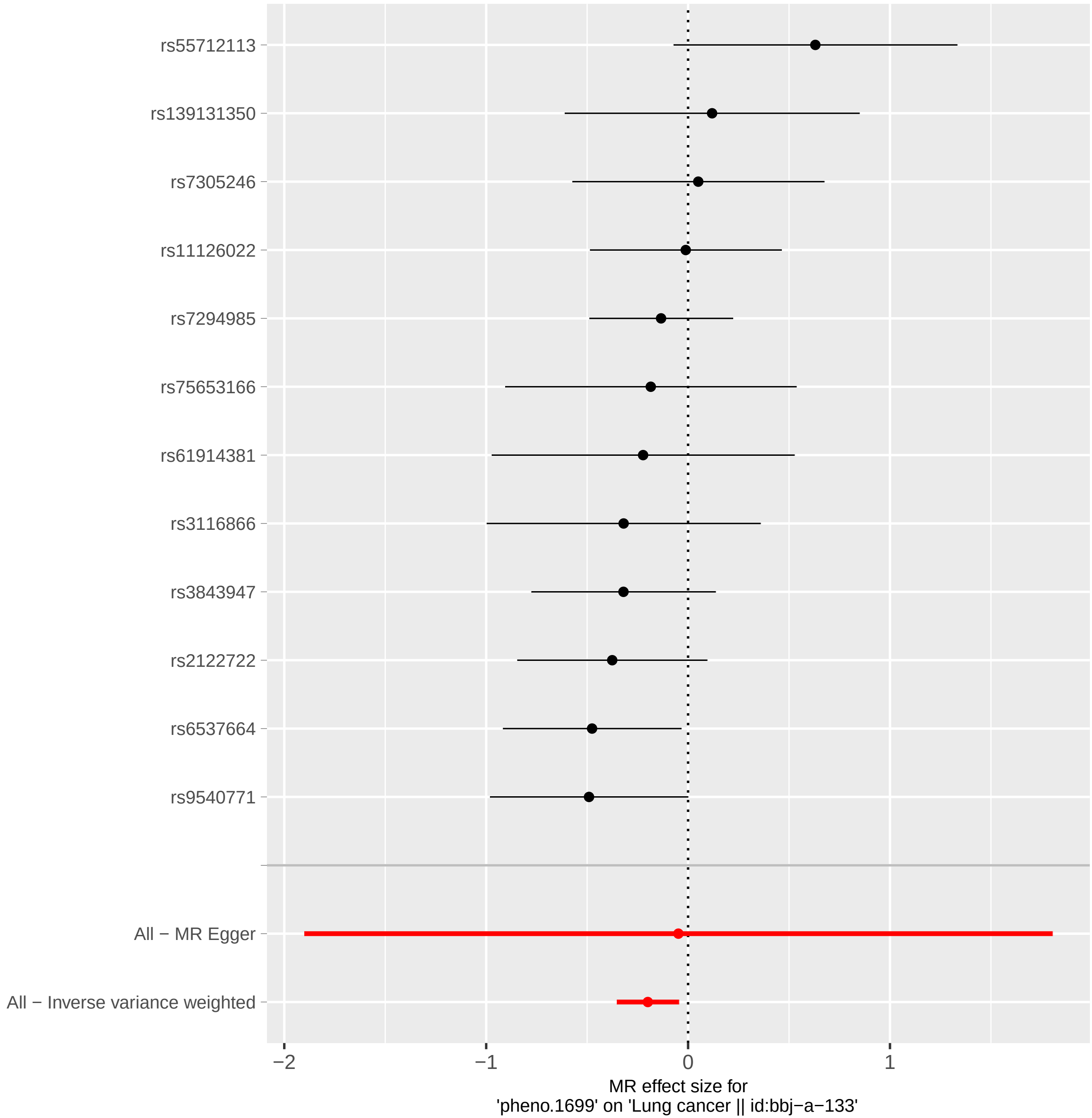

rs55712113
rs139131350
rs7305246
rs11126022
rs7294985
rs75653166
rs61914381
rs3116866
rs3843947
rs2122722
rs6537664
rs9540771
All − MR Egger
All − Inverse variance weighted
−2
−1
0
1
MR effect size for
'pheno.1699' on 'Lung cancer || id:bbj−a−133'
